# ﻿ *Primulaxinningensis* (Primulaceae), a new species from karst caves in Hunan, China

**DOI:** 10.3897/phytokeys.199.85231

**Published:** 2022-06-13

**Authors:** Wei Zhang, Yu Zhang, Jian Wen Shao

**Affiliations:** 1 College of Life Sciences, Anhui Normal University, Wuhu, Anhui 241000, China Anhui Normal University Wuhu China; 2 Provincial Key Laboratory of Conservation and Utilization of Biological Resources, Wuhu, Anhui 241000, China Provincial Key Laboratory of Conservation and Utilization of Biological Resources Wuhu China

**Keywords:** Homostyly, *P.cicutariifolia*, *P.hubeiensis*, *P.merrilliana* complex, section *Ranunculoides*

## Abstract

*Primulaxinningensis* Wei Zhang bis & J.W.Shao, a new species from Hunan Province, China, is described. Its leaf morphology is similar to the *P.merrilliana* complex and flower morphology similar to *P.cicutariifolia*, but it can be distinguished from the former by the black pollen sac, corolla lobes apex obviously emarginate and can be differed from the latter by cotyledon triangular obovate, plants densely covered with glandular hairs and special habitat (karst caves). The whole plastid genome of this new species is 151, 601–151, 630 bp in length. Based on the whole plastid genome sequences, phylogenetic trees revealed that the new species did not genetically relate to the above two mentioned morphologically similar species, but it was closely related to *P.hubeiensis*. Currently, only three populations were discovered within a small distribution area, thus, it is preliminarily considered as Vulnerable (VU) according to criteria of the IUCN Red List.

## ﻿Introduction

*Primula* L. is the largest genus in Primulaceae and comprises about 500 species worldwide. The genus is mainly distributed in temperate and alpine regions of the Northern Hemisphere, with only a few species in the Southern Hemisphere, i.e. Africa, tropical Asia and South America (Richards 2002). There are approximately 300 native *Primula* species in China and the modern distribution centres of this genus are located along both sides of the Himalayas to Yunnan and western Sichuan (Hu and Kelso 1996).

The sect. Ranunculoides C.M.Hu is a unique group in *Primula*, characterised by pinnately compound leaves and calyx not inflated at the base (Hu 1990; Hu and Kelso 1996). This section now includes *P.ranunculoides* F.H.Chen, *P.cicutariifolia* Pax, *P.jiugongshanensis* J.W.Shao, *P.hubeiensis* X.W.Li and *P.merrilliana* complex (Shao et al. 2012; He et al. 2017; Li et al. 2018; He et al. 2021). They are all endemic to central or eastern China and often grow at the waterside or the edge of broadleaf deciduous forests between 50 and 1600 m (Shao et al. 2012; He et al. 2017; Zhang et al. 2021).

In March 2016, during our field expeditions in Shimen Village, Xinning County, Hunan Province, China, we encountered a suspicious species of sect. Ranunculoides. The plants were restricted to growing on the walls and ground near the entrance to karst caves and are quite different from other known related species. After careful morphological observations, together with evidence from molecular phylogenetic analyses, based on the chloroplast genome, this suspicious species was confirmed as a new species. Here, the investigation results are reported, the new species is named as *Primulaxinningensis* Wei Zhang bis & J.W.Shao and is described.

## ﻿Materials and methods

### ﻿Sampling and morphological analyses

The studied specimens were collected in Shimen Village (26°30'27.22"N, 110°40'56.82"E, altitude: 468 m), Xinning County, Hunan Province, China. Voucher specimens were deposited at the Herbarium of Anhui Normal University (ANUB). The morphological description of the new species was based on examination of fresh material and herbarium specimens. A total of 10 diagnostic characteristics of the new species were identified and compared to related species in the Primulasect.Ranunculoides (Shao et al. 2012; He et al. 2017; Li et al. 2018; He et al. 2021).

### ﻿Genome sequencing, assembly and annotation

Genomic DNA was extracted from dried leaves using a modified CTAB protocol (Doyle and Doyle 1987). The quality and concentration of DNA products were assessed via agarose gel electrophoresis and spectrophotometry and the qualified DNA sample was sent to BGI-Shenzhen (Shenzhen, China) for library construction and next-generation sequencing. Finally, we obtained ca. 2 Gb of high-quality clean data, the complete chloroplast genome was assembled using GetOrganelle described in Jin et al. (2019) and the annotation was conducted with Plastid Genome Annotator (Qu et al. 2019), coupled with manual correction using Geneious v. 9.1.4 (Kearse et al. 2012). The plastome of *P.hubeiensis* (Genbank accession number: MT268976) was used as the reference genomes for annotation. The cp genome maps were drawn using OGDRAW (Greiner et al. 2019). All sequences generated in this study were submitted to the NCBI database, the accession numbers are ON208991 (*P.xinningensis*), ON208990 (*P.xinningensis*) and ON221323 (*P.hubeiensis*), respectively.

### ﻿Phylogenetic analyses

In order to determine the phylogenetic relationship of the new species, we downloaded 28 accessions cp genome sequences of *primula* from the NCBI (Fig. 2). All sequences were aligned with MAFFT v.7 (Katoh and Standley 2013) using the default settings and adjusted manually where necessary using MEGA 7.0.14 (Kumar et al. 2016). Phylogenetic analyses were conducted using Maximum Likelihood (ML) and Bayesian Inference (BI) methods with *Androsacepaxiana* and *Lysimachiacongestiflora* as outgroups (Xu et al. 2020). The ML analysis was conducted using RAxML-HPC BlackBox v.8.1.24 at the CIPRES Science Gateway website (Miller et al. 2010; Stamatakis 2014) with 1000 bootstrap replicates, the (GTR) + G + I model being used in ML analyses. For the BI analysis, the best substitution model was determined according to Bayesian Information Criterion (BIC) with ModelFinder (Kalyaanamoorthy et al. 2017). The BI analysis was performed using MrBayes v.3.2 (Ronquist et al. 2012). The Markov Chain Monte Carlo (MCMC) algorithm was run for 10 million generations and the trees were sampled every 1000 generations. Convergence was determined by examining the average standard deviation of the split frequencies (< 0.01). The first 25% of the trees were discarded as burn-in and the remaining trees were used to generate the consensus tree.

## ﻿Results

### ﻿Characteristics of the complete plastid genome

**Figure 1.**
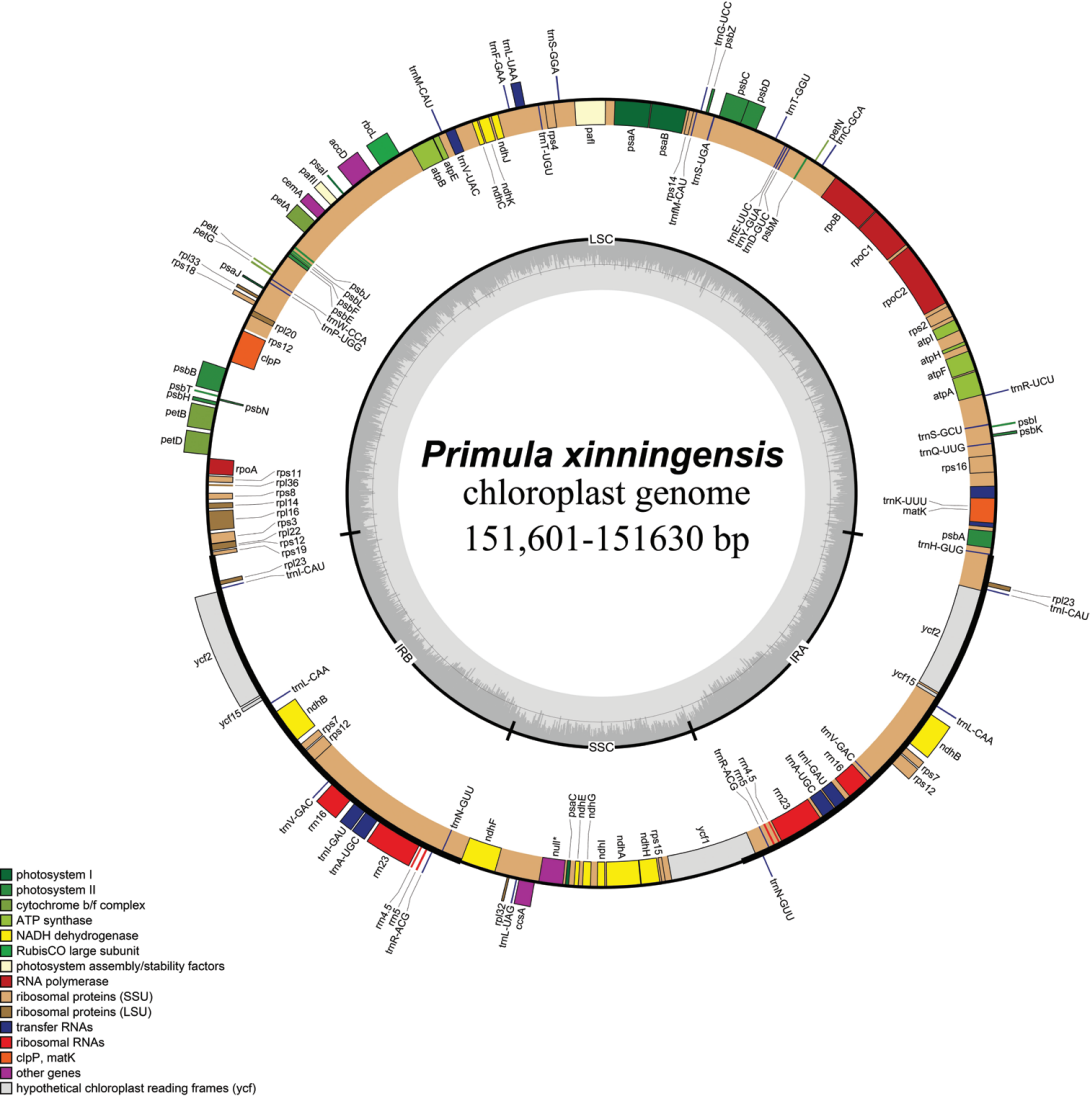
Plastid genome map of *P.xinningensis* sp. nov.

The length of complete plastid genome of *P.xinningensis* comprised 151,601–151,630 bp (Fig. 1). It possessed typical quadripartite structure: IRa, IRb, LSC and SSC; the characteristics and statistics of the plastid genome are summarised in Table 1.

**Table 1.** Basic characteristics of cp genomes of *Primulaxinningensis* sp. nov.

Characteristic	*Primulaxinningensis*
Total length (bp)	151,601–151,630
GC%	36.8%–36.8%
LSC length (bp)	83,421–83,466
SSC length (bp)	17,583–17,599
IR length (bp)	25,292
Total genes	113
Protein-coding genes	80
rRNA genes	4
tRNA genes	29

### ﻿Molecular phylogenetic relationship

**Figure 2.**
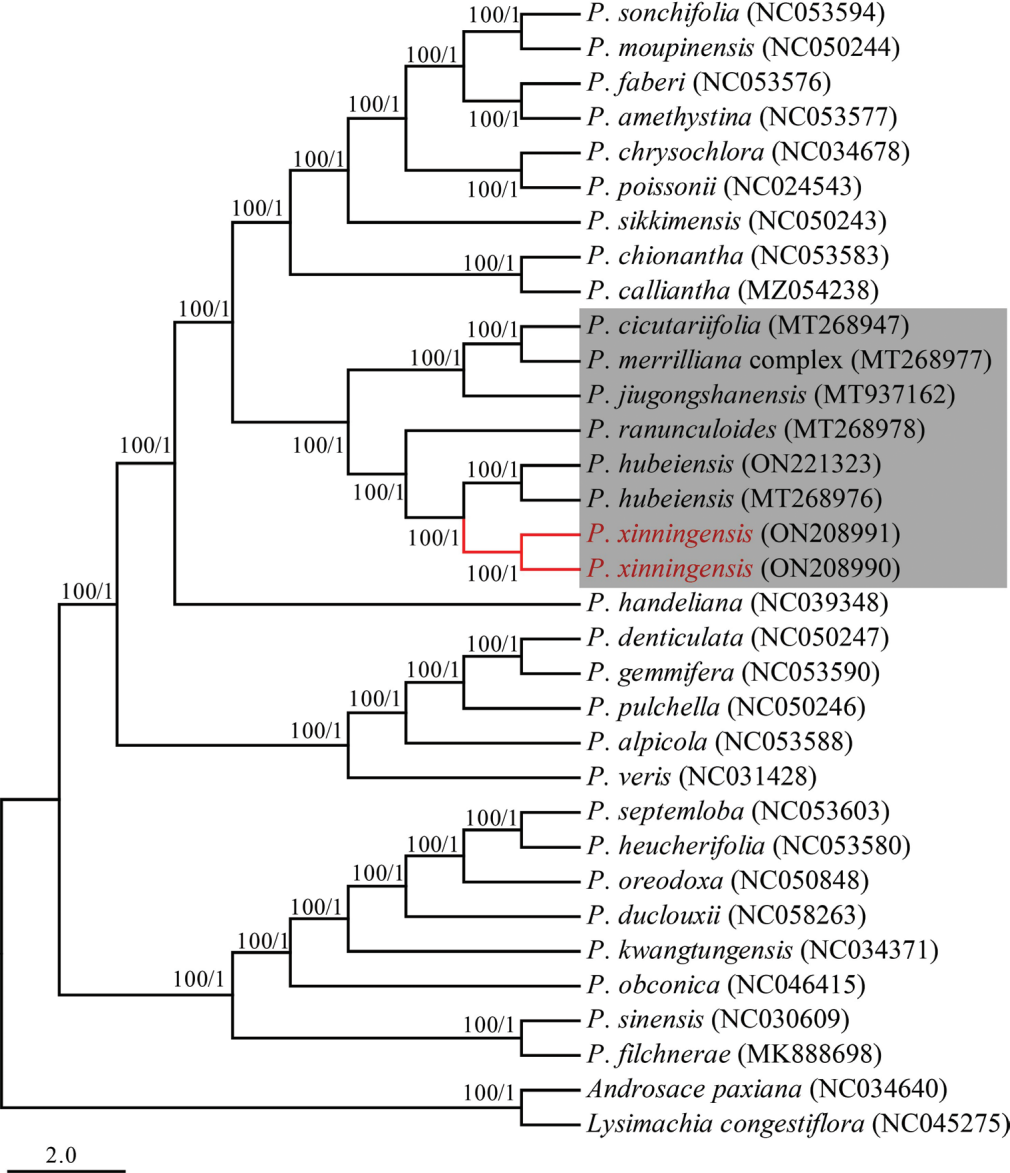
Phylogenetic relationships of *P.xinningensis* sp. nov. and related species inferred from ML and BI analyses, based on the whole plastid genome. Numbers on the branches indicate the bootstrap support of the ML and the posterior probability of BI analyses. NCBI accession numbers were shown in the parentheses.

Phylogenetic relationships of the new species and related species were constructed, based on the whole plastid genome using ML and BI analyses. The results showed that *P.xinningensis* affiliate to sect. Ranunculoides. In sect. Ranunculoides, *Primulamerrilliana* complex, *P.cicutariifolia* and *P.jiugongshanensis* clustered in one clade and the other three species (*P.xinningensis*, *P.hubeiensis* and *P.ranunculoides*) clustered in another clade (Fig. 2). *Primulaxinningensis* is a sister species of *P.hubeiensis*, and their individuals were respectively grouped into a monophyly with high support (posterior probability (PP) = 1, bootstrap support (BS) = 100%) (Fig. 2).

### ﻿Morphological comparison

**Figure 3.**
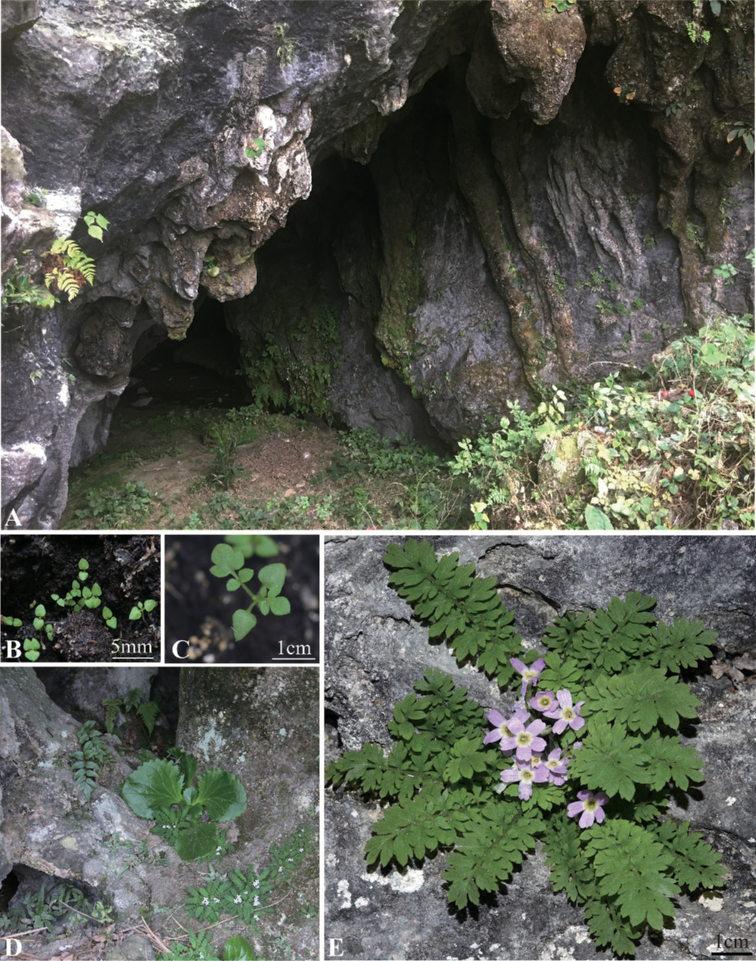
Living images of *P.xinningensis* sp. nov. **A, D** habitat **B, C** plant in seedling **E** plant in ﬂowering.

**Figure 4.**
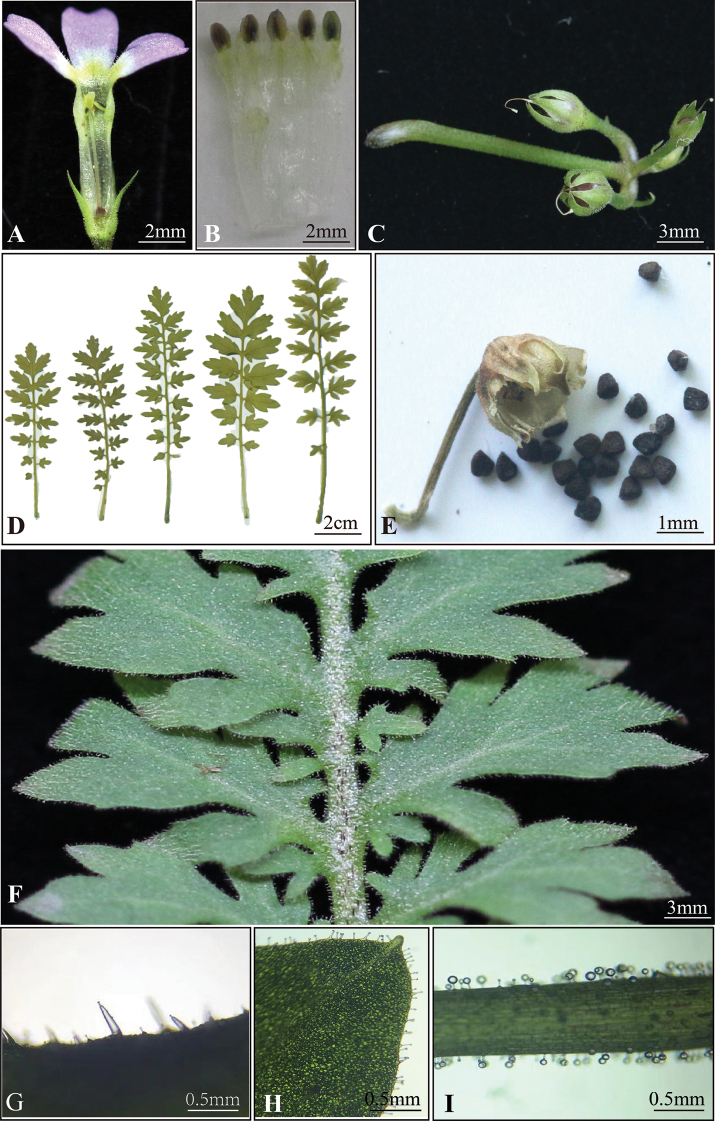
Morphological characters of *P.xinningensis* sp. nov. **A, B** longitudinally dissected of floral tube **C** infructescence **D, F** leaves morphology **E** opened capsule and seeds **G** leaf surface glandular hairs **H** leaf margin glandular hairs **I** rachis glandular hairs.

In morphology, this new species is very similar to *P.merrilliana* complex in leaf pinnae shape and degree of division and similar to *P.cicutariifolia* in floral characters, but can be easily distinguished from the former by the black pollen sac, corolla lobes apex obviously emarginate and can be differed from the latter by cotyledon triangular obovate, pinna margin usually pinnatipartite and plants densely covered with glandular hairs (Table 2, Figs 3, 4). Although, in the phylogenetic relationship, *P.xinningensis* is closely related to *P.hubeiensis*, there were obvious morphological differences between them in pinna division pattern (segments margin entire vs. segments margin serrate), the length of glandular hair (0.07–0.42 mm vs. 0.76–0.88 mm) and flowers size and type (homostylous and corolla diameter 8–12 mm vs. distyly and corolla diameter 13–18 mm) (Table 2, Figs 3, 4). Detailed morphological comparisons between the new species and other related species in sect. Ranunculoides are summarised in the following key:

**Table 2.** Morphological and ecological features comparison between *P.xinningensis* sp. nov. and its related species.

Features	*P.xinningensis*	*P.hubeiensis*	*P.cicutariifolia*	*P.merrilliana* complex
Floral morph	Homostylous	Distylous	Homostylous	Distylous or homostylous
Umbel layers	1	1–2	1	1–3
Corolla diameter	8–12 mm	13–18 mm	6–10 mm	9–19 mm
Corolla lobes	Apex conspicuously emarginate	Apex conspicuously emarginate	Apex conspicuously emarginate	Apex rounded
Scape length	0.8–2 cm	3.5–9.6 cm	1–3 cm	1.3–9 cm
Pollens	Pantoporate	Pantoporate	Pantoporate	Pantoporate or stephanocolpate
Pollen sac	Black	Yellow	Yellow	Yellow
Cotyledon	Triangular obovate	Ovate	Ovate	Ovate
Older Leaves	Pinnatisect, with 11–19 pinnae, the terminal pinna similar to others, 3-lobed or parted	Pinnatisect, with 13–19 pinnae, the terminal pinna similar to others, 3-lobed or parted, margin coarsely dentate	Pinnatisect, with 7–17 pinnae, the terminal pinna similar to others, 3-lobed	Pinnatisect, with 11–21 pinnae, the terminal pinna similar to others, 3-lobed or parted
Glandular hairs	Leaves and scape densely covered with glandular hairs (0.07–0.42 mm)	Leaves and scape densely covered with glandular hairs (0.76–0.88 mm)	without	without
Distribution	Hunan	Hubei	Anhui, Zhejiang	Anhui, Zhejiang
Habitat	Karst caves	Shady damp rock crevices	Stream sides or under broadleaf deciduous forests of northern slopes	Stream sides or under broadleaf deciduous forests of northern slopes

### ﻿Key to the species of sect. Ranunculoides

**Table:** 

1	Corolla lobe apices rounded	***P.merrilliana* complex**
–	Corolla lobe apices obviously emarginated	**2**
2	Compound leaves with 3–9 pinnae; scape apices diﬀerentiating to bulblets late in ﬂowering	***P.ranunculoides***
–	Compound leaves with 7–21 pinnae; scape apices lacking bulblets	**3**
3	Plants densely covered with glandular hairs	**4**
–	Plants glabrous	**5**
4	Cotyledon triangular obovate, pinna segments margin entire, flower homostylous, corolla diameter 8–12 mm	***P.xinningensis***
–	Cotyledon ovate, pinna segments margin serrate, flower distylous, corolla diameter 13–18 mm	***P.hubeiensis***
5	Flower homostylous, umbels solitary, limb ca. 6–10 mm across	***P.cicutariifolia***
–	Flower distylous, umbels usually 2, limb ca.11–19 mm across	***P.jiugongshanensis***

### ﻿Taxonomic treatment

#### ﻿ Primula xinningensis

Taxon classificationPlantaeEricalesPrimulaceae

Wei Zhang bis & J.W.Shao sp. nov.

55FA90FF-610E-5FFD-BB0F-C2569B64CCDD

urn:lsid:ipni.org:names: 7299424-1

Figs 3 , 4 , 5

##### Type.

**Figure 5.**
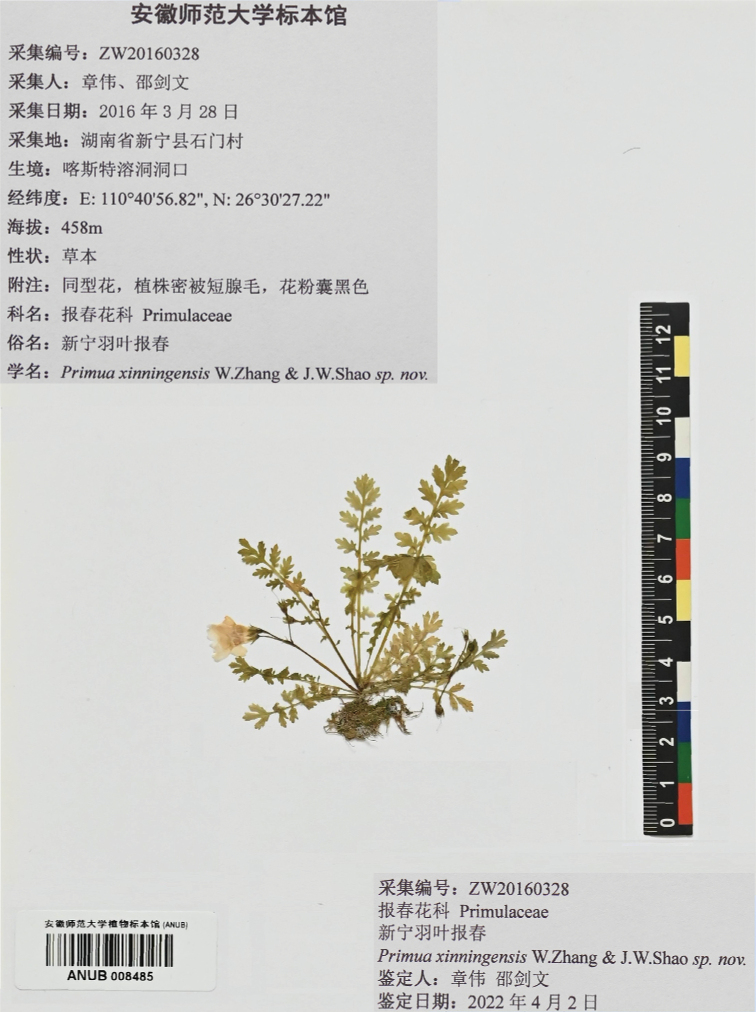
Holotype of *P.xinningensis* Wei Zhang bis & J.W.Shao, sp. nov.

China. Hunan, Xinning County, Shimen Village, 26°30'27.22"N, 110°40'56.82"E, alt. 458 m, 28 Mar 2016, *Wei.Zhang & Jian.Wen.Shao* ZW20160328 (holotype: ANUB!; isotypes: ANUB!, PE!) (Fig. 5).

##### Diagnosis.

Cotyledon triangular obovate, leaves and scape densely covered with short glandular hairs, flowers long homostyled, corolla lobe apices conspicuously emarginate, pollen sac black.

##### Description.

Herb biennial, dwarf, densely covered with short glandular hairs. Leaves 9–25 in an open rosette; petiole 1.0–2.0 cm long; leaf blade pinnatisect, 4.0–8.0 cm long, 1.0–2.0 cm wide; pinnae 5–9 pairs, elliptic, margin usually pinnatifid, segments 3–5, apex mucronulate. Scapes 1–8 in each plant, 0.8–2.0 (–2.5) cm tall, carrying one umbel, usually 3 flowers per umbel; bracts linear lanceolate, 2–3 mm long. Pedicel slender, 0.3–0.8 cm. Flowers long homostylous. Calyx narrowly campanulate, 1.0–3.0 mm long, split to the middle; lobes lanceolate, apex acuminate, not outward curvature. Corolla pale red, flowers’ limb 8.0–12.0 mm across, lobes obovate, ca. 2.5 mm wide, apex conspicuously emarginate, corolla tube 5.8–7.0 mm, both stamens and stigma at the mouth of the corolla tube, pollen sac black. Capsule subglobose, 1.0–3.0 mm in diam., dehiscing by valves.

##### Phenology.

Flowering from March to April, fruiting from April to May.

##### Chinese name.

Xīn níng yǔ yè bào chūn (新宁羽叶报春).

##### Etymology.

The specific epithet ‘*xinningensis*’ refers to the locality, Xinning County, Hunan, China.

##### Distribution and ecology.

*P.xinningensis* is known only from Shimen Village, Xinning County, Hunan, China. Growing on the walls or ground of karst caves, at an altitude of 385–487 m. The main accompanied species were *Primulinalatinervis* (W.T.Wang) Mich. Möller & A. Weber. (Gesneriaceae), *Cyrtomiumfortunei* J.Sm. (Dryopteridaceae) and *Pterismultifida* Poir. (Pteridaceae).

##### Conservation status.

Vulnerable (VU D1 and D2). This species is endemic to China, Hunan Province, Xinning County, Shimen Village. We only found three populations, all of them near the entrance to the karst caves and each with about 100–250 individuals. The surrounding area is cultivated field with strong human activities. The Extent of Occurrence (EOO) is less than 10 km^2^ and the known Area of Occupancy (AOO) is less than 0.5 km^2^. Therefore, the conservation status of this new species is evaluated as ‘Vulnerable’ (VU) as it meets criterion D1 and D2, according to the IUCN Red List Categories and Criteria (IUCN 2019). In addition, *P.shimemensis* is a homostylous species. Although *Primula* containing ca. 500 species, there are only ca. 45 species having monomorphic populations (Mast et al. 2006). Therefore, the recognition of this new species increases the homostylous species diversity in *Primula* and can provide valuable material for studying the evolution and maintenance mechanism of distylous flowers.

##### Additional specimen examined.

China. Hunan: Xinning County, Wanfeng Forest Farm, alt. 450 m, 22 Apr 1995, *Lin Bo Luo* 00205565 (PE); Xinning County, Wanfeng Forest Farm, alt. 450 m, 22 Apr 1995, *Lin Bo Luo* 00353811 (IBK); Xinning County, Shuimiao Town, Jiangmu Village, alt. 347 m, 19 Feb 2014, *Xun Lin Yu & Hui Zhou* 028303 (CSFI); Xinning County, Shuimiao Town, Jiangmu Village, alt. 347 m, 19 Feb 2014, *Xun Lin Yu & Hui Zhou* 028304 (CSFI).

## Supplementary Material

XML Treatment for Primula xinningensis

## References

[B1] Doyle JJ Doyle JL (1987) A rapid DNA isolation procedure for small quantities of fresh leaf tissue. Phytochemical Bulletin 19(1): 11–15.

[B2] Greiner S Lehwark P Bock R (2019) OrganellarGenomeDRAW (OGDRAW) version 1.3.1: Expanded toolkit for the graphical visualization of organellar genomes. Nucleic Acids Research 47(1): 59–64. 10.1093/nar/gkz238 PMC6602502 30949694

[B3] He X Song LY Wu YF Liu J Shao JW (2017) *Primulajiugongshanensis* sp. nov. (Primulaceae) from China, based on morphological and molecular evidence. Nordic Journal of Botany 35(3): 328–333. 10.1111/njb.01471

[B4] He X Cao JJ Zhang W Li YQ Zhang C Li XH Shao JW (2021) Integrative taxonomy of herbaceous plants with narrow fragmented distributions: A case study on *Primulamerrilliana* species complex. Journal of Systematics and Evolution (early view). 10.1111/jse.12726

[B5] Hu CM (1990) *Primula*. In: Chen FH Hu CM (Eds) Flora Reipublicae Popularis Sinicae, Chapter 59.Science Press, Beijing, 1–245.

[B6] Hu CM Kelso S (1996) Primulaceae. In: Wu ZY, Raven PH (Eds) Flora of China. Science Press & Missouri Botanical Garden Press, Beijing & St. Louis.

[B7] IUCN (2019) Guidelines for Using the IUCN Red List Categories and Criteria. Version 14. Prepared by the Standards and Petitions Committee. http://www.iucnredlist.org/documents/RedListGuidelines.pdf [accessed 4 Sep 2019]

[B8] Jin JJ Yu WB Yang JB Song Y Li DZ (2019) GetOrganelle: A fast and versatile toolkit for accurate de novo assembly of organelle genomes. Genome Biology 21(1): e241. 10.1186/s13059-020-02154-5 PMC7488116 32912315

[B9] Kalyaanamoorthy S Minh BQ Wong T von Haeseler A Jermiin LS (2017) ModelFinder: Fast model selection for accurate phylogenetic estimates. Nature Methods 14(6): 587–589. 10.1038/nmeth.4285 28481363 PMC5453245

[B10] Katoh K Standley DM (2013) MAFFT multiple sequence alignment software version 7: Improvements in performance and usability. Molecular Biology and Evolution 30(4): 772–780. 10.1093/molbev/mst010 23329690 PMC3603318

[B11] Kearse M Moir R Wilson A Stones-Havas S Cheung M Sturrock S Buxton S Cooper A Markowitz S Duran C Tierer T Ashton B Meintjes P Drummond A (2012) Geneious Basic: An integrated and extendable desktop software platform for the organization and analysis of sequence data. Bioinformatics (Oxford, England) 28(12): 1647–1649. 10.1093/bioinformatics/bts199 22543367 PMC3371832

[B12] Kumar S Stecher G Tamura K (2016) MEGA7: Molecular evolutionary genetics analysis version 7.0 for bigger datasets. Molecular Biology and Evolution 33(7): 1870–1874. 10.1093/molbev/msw054 27004904 PMC8210823

[B13] Li XW Bao DC Huang HD Xie JF (2018) *Primulahubeiensis* (primulaceae), a new species from central china. Novon A Journal for Botanical Nomenclature 25(2): 162–165. 10.3417/2016032

[B14] Mast AR Kelso S Conti E (2006) Are any primroses (*Primula*) primitively monomorphic? The New Phytologist 171(3): 605–616. 10.1111/j.1469-8137.2006.01700.x 16866962

[B15] Miller MA Pfeiffer W Schwartz T (2010) Creating the CIPRES Science Gateway for inference of large phylogenetic trees. 2010 Gateway Computing Environments Workshop (GCE), 1–8. 10.1109/GCE.2010.5676129

[B16] Qu XJ Moore MJ Li DZ Yi TS (2019) PGA: A software package for rapid, accurate, and flexible batch annotation of plastomes. Plant Methods 15(1): e50. 10.1186/s13007-019-0435-7 PMC6528300 31139240

[B17] Richards A (2002) *Primula* (2^nd^ edn.). B.T. Batsford, London.

[B18] Ronquist F Teslenko M van der Mark P Ayres DL Darling A Höhna S Larget B Liu L Suchard MA Huelsenbeck JP (2012) MrBayes 3.2: Efficient Bayesian phylogenetic inference and model choice across a large model space. Systematic Biology 61(3): 539–542. 10.1093/sysbio/sys029 22357727 PMC3329765

[B19] Shao JW Wu YF Kan XZ Liang TJ Zhang XP (2012) Reappraisal of *Primularanunculoides*, (Primulaceae), an endangered species endemic to China, based on morphological, molecular genetic and reproductive characters. Botanical Journal of the Linnean Society 169(2): 338–349. 10.1111/j.1095-8339.2012.01228.x

[B20] Stamatakis A (2014) RAxML version 8: A tool for phylogenetic analysis and post-analysis of large phylogenies. Bioinformatics (Oxford, England) 30(9): 1312–1313. 10.1093/bioinformatics/btu033 24451623 PMC3998144

[B21] Xu WB Xia BS Li XW (2020) The complete chloroplast genome sequences of five pinnate-leaved *Primula* species and phylogenetic analyses. Scientific Reports 10(1): e20782. 10.1038/s41598-020-77661-3 PMC7699626 33247172

[B22] Zhang W Hu YF He X Zhou W Shao JW (2021) Evolution of autonomous selfing in marginal habitats: Spatiotemporal variation in the floral traits of the distylous *Primulawannanensis*. Frontiers in Plant Science 12: e781281. 10.3389/fpls.2021.781281 PMC8716950 34975966

